# Self-Removal of Medical Devices in the ICU: A Retrospective Study

**DOI:** 10.7759/cureus.86865

**Published:** 2025-06-27

**Authors:** John Culhane, Raymond Okeke, Kellie Bushe, Carl Freeman

**Affiliations:** 1 Trauma, SSM Health St. Louis University Hospital, St. Louis, USA; 2 Trauma, Saint Louis University School of Medicine, St. Louis, USA

**Keywords:** extubation, icu mortality, line removal, medical devices, self-removal, srmd

## Abstract

Introduction

Self-removal of medical devices (SRMD) is common in the intensive care unit (ICU). Most studies of this issue concentrate on self-extubation, leaving self-removal of other devices less well studied.

Methods

This is a retrospective chart review utilizing the MIMIC III database. Free-text notes were examined for reports of patients removing medical devices. Predictive factors and the outcome of mortality were analyzed. Univariate analysis of categorical variables was performed using chi-square and continuous variables using the t-test. Multivariate analysis was performed with logistic regression. Covariates were gender, age, tobacco abuse, delirium, alcohol abuse, psychiatric history, drug abuse, dementia, and brain trauma.

Results

Overall, 5.3% of ICU patients pulled at least one device. The number of devices pulled per 1,000 ICU days was 21.5. The devices most pulled were nasogastric tubes (11.87), peripheral intravenous catheters (3.1), and Foley catheters (2.0). Death was more likely for patients who removed devices: 56% versus 40% (p<0.001). SRMD was an independent risk factor for death, with an adjusted odds ratio (OR) of 1.9 (p<0.001).

On univariate analysis, a history of delirium was the most substantial predictive factor at 16.3%, followed by a history of alcohol withdrawal (16.2%) and alcohol abuse (9.9%), p<0.001 for all comparisons. On multivariate analysis, history of delirium remained the most decisive independent risk factor with an OR of 3.15, followed by a history of alcohol withdrawal (OR 2.0) and a history of drug abuse (OR 1.7), p<0.001 for all comparisons.

Conclusions

SRMD occurs with clinically important frequency in the ICU. Nasogastric tubes are poorly tolerated. SRMD is an independent predictor of ICU mortality. Delirium plays a central role in SRMD, with alcohol use and drug use as additional essential factors. This suggests a focus on delirium control as the best way to prevent SRMD and improve patient safety.

## Introduction

Iatrogenic self-removal of medical devices (SRMD) is a challenging problem in the intensive care unit (ICU). Self-removal of devices such as nasogastric tubes, intravenous catheters, and indwelling urinary catheters results in the immediate loss of the device's intended function, often leading to therapy interruption. It may also cause serious patient harm due to several other mechanisms, including tissue damage, bleeding, and the need for replacement with associated procedural risks. SRMD is also associated with longer-term sequelae such as prolonged hospital stay, increased need for chronic care, and increased mortality [1,2].

Preventing self-removal is the cornerstone of avoiding these risks. Various factors contribute to the inadvertent removal of medical devices. There is a significant gap in the understanding of the specific characteristics of patients that lead to this phenomenon [3]. Identifying these characteristics is crucial, as it would enable healthcare providers to address modifiable factors. By using these characteristics to identify vulnerable patients, interventions can be directed toward those at high risk, reducing the occurrence of SRMD and preventing adverse patient outcomes. This study aims to create a profile of vulnerable patients, facilitating targeted interventions and enhancing device-related safety. The secondary aim of the study is to investigate how device self-removal predicts outcomes, providing insights into how securing devices can improve patient safety.

## Materials and methods

This retrospective registry review seeks to quantify and correlate SRMD with variables derived from demographic data, medical history, and day-to-day patient care. The study population is a mixed medical and surgical ICU cohort from the MIMIC III database. MIMIC III is a public registry of ICU data from Beth Israel Deaconess Hospital in Boston, MA. Although MIMIC III is not the most recent version of this database, it was chosen because it includes all daily progress notes. The inclusion criteria were patients aged 17 and above. The only exclusion criteria were related to inadequate documentation of the circumstances of device self-removal. The thoroughness of our methods instills confidence in the robustness of our research.

Demographics were retrieved from the abstracted portion of the database. Further study parameters were derived from free-text notes. The notes were examined with software developed at our institution. Instances of patients removing their own invasive devices were recorded. The devices included were arterial catheters, peripheral venous catheters, central venous catheters including those peripherally inserted, various wires such as pacemaker wires, nasogastric and nasoenteric tubes, gastrostomy tubes both endoscopically and surgically placed, indwelling urinary catheters, and surgical drains. Endotracheal tubes were excluded.

Predictive factors for device removal were analyzed. For variables unlikely to change during admission, such as demographics and chronic conditions, the device removal rate was recorded per patient. For variables more subject to daily variation, such as pain levels and extubation status, the removal rate was recorded per patient day. The authors analyzed the association of device removal and covariates with mortality.

Statistical analysis was performed as follows. Sample size calculations were omitted because the entire database was queried. Statistical power was considered adequate due to multiple highly significant results, making type 2 errors unlikely. Univariate analysis of categorical variables was performed using chi-square and continuous variables using the t-test. Multivariate analysis was performed with logistic regression. Covariates were gender, age, tobacco abuse, delirium, alcohol abuse, psychiatric history, drug abuse, dementia, and brain trauma.

## Results

Our study included 38,650 patients comprising more than 153,466 ICU days. Overall, 5.3% of patients pulled at least one device. Nasogastric tubes were removed at the highest rate, followed by peripheral intravenous catheters and Foley catheters. Gastrostomy tubes and central venous catheters, devices whose unplanned removal is potentially most dangerous, were pulled at a lower rate of about one per 1,000 ICU days (Table 1).

**Table 1:** Rate of self-extraction per device Nasogastric tubes were removed at by far the highest rate, followed by peripheral IV catheters and Foley catheters.

Device	Patients who pulled a device	Devices pulled	Devices pulled per 1,000 ICU days
Any device	2,062	3,299	21.50
Nasogastric tubes	1,369	1,821	11.87
Peripheral IV catheters	410	475	3.10
Foley catheters	232	301	1.96
Arterial lines	191	205	1.34
Gastrostomy tubes	126	145	0.94
Central lines	124	139	139
PICC	99	113	0.74
Surgical drains	54	66	0.43
Various wires	23	24	0.16

After examining total patients with self-extraction, patients were evaluated according to their admitting critical care unit: CCU (coronary care unit), CSRU (cardiac surgery recovery unit), MICU (medical intensive care unit), SICU (surgical intensive care unit), and TSICU (trauma surgical intensive care unit). Device extraction rates for MICU, SICU, and TSICU (trauma) are nearly equal at about 6% and those for CCU and CSRU are lower (p<0.001 for any difference between groups). The mean age of patients who pulled any device was 65.5 versus 64.0 for those who did not pull a device (p<0.001) (Table 2).

**Table 2:** Device self-extraction by the intensive care unit to which the patient was first admitted CCU, coronary care unit; CSRU, cardiac surgery recovery unit; MICU, medical intensive care unit; SICU, surgical intensive care unit; TSICU, trauma surgical intensive care unit

Unit	n (%)
MICU	925 (6.5)
SICU	383 (6.0)
TSICU	315 (6.1)
CCU	238 (4.4)
CSRU	201 (2.7)

Many demographic and medical history factors are associated with device self-removal. Some characteristics such as delirium may be part of both the documented medical history and daily assessment. The context in the free-text note was used to decide the status for each instance. Since this is a retrospective chart review, data representing every variable was not necessarily recorded for every patient. Numbers for binary variables may not add up to the entire patient population. The percentage is the percent of patients who pulled a device when the condition was present versus when it was absent. The mean CIWA (Clinical Institute Withdrawal Assessment for Alcohol) score in patients who removed any device was 12.5 versus 9.8 for those with no recorded device self-removal (p=0.01) (Table 3).

**Table 3:** Univariate analysis of patient level predictors of device self-removal A p-value of ≤0.05 was considered significant

Characteristic	Present, n (%)	Absent, n (%)	p-value
History of delirium	413 (16.3)	1649 (4.6)	<0.001
History of alcohol withdrawal	151 (16.2)	1911 (5.1)	<0.001
History of alcohol abuse	327 (9.9)	1735 (4.9)	<0.001
Psychiatric history	1014 (7.6)	1048 (4.1)	<0.001
History of traumatic brain injury	139 (7.8)	1923 (5.2)	<0.001
Dementia	92 (9.5)	1970 (5.2)	<0.001
Male gender	1230 (5.6)	832 (5.0)	0.004
Non-English speaking	78 (5.2)	710 (4.1)	0.04
History of tobacco abuse	266 (4.9)	1796 (5.4)	0.10

Once commonalities such as demographic and history factors were recognized, a univariate analysis of patient-day level (acutely changing) predictors of device self-removal was conducted, considering the Glasgow Coma Scale, CIWA, need for a sitter, disorientation, pain, and use of restraints. The percentage calculated was the percent of patient days on which a device was pulled when the condition was present versus when it was absent (Table 4).

**Table 4:** Association of device self-removal with factors that vary day-to-day A p-value of ≤0.05 was considered significant. N refers to patient days with the condition present. CIWA, Clinical Institute Withdrawal Assessment for Alcohol; GCS, Glasgow Coma Scale

Characteristic	Present, n (%)	Absent, n (%)	p-value
Sitter	151 (5.9)	2069 (0.7)	<0.001
CIWA positive	39 (3.2)	19 (1.7)	0.02
Disoriented	949 (2.9)	452 (0.5)	<0.001
GCS<15 (extubated)	1059 (2.1)	177 (0.4)	<0.001
Restrained	1373 (1.6)	389 (0.8)	<0.001
Any pain	214 (0.7)	1901 (1.0)	<0.001
Pain greater than moderate pain	202 (0.6)	391 (0.7)	0.04
Ventilated	447 (0.5)	1781 (0.9)	<0.001

Table 5 shows the multivariate analysis of the outcome of death associated with device self-removal and other covariates. Self-removal of any device is the strongest binary predictor of death versus gender and other behavioral variables.

**Table 5:** The odds ratio of mortality associated with device self-removal and other covariates A p-value of ≤0.05 was considered significant

Characteristic	OR	p-value
Self-extraction of any device	1.95	<0.001
Dementia	1.43	<0.001
History of alcohol abuse	1.18	<0.001
Age	1.05	<0.001
Tobacco abuse	0.75	<0.001
History of drug abuse	1.09	0.33
Psychiatric history	0.99	0.71
Male gender	0.98	0.40
History of delirium	0.97	0.49
Traumatic brain injury	0.90	0.06

Table 6 shows that mental status changes, whether acute or based on medical history, seem to be the strongest predictors of SRMD. On multivariate analysis, age and all behavior-related variables except dementia were significant independent predictors of device self-removal. Delirium was by far the strongest positive predictor, with adjusted odds of self-removal 3.15 times higher for delirious versus non-delirious patients (Table 6). Mortality for patients who pulled any device was 1,153 (56%) versus 14,540 (40%) for those who did not pull a device (p<0.001).

**Table 6:** The adjusted odds ratios of selected predictors of device self-removal A p-value of ≤0.05 was considered significant

Characteristic	OR	p-value
History of delirium	3.15	<0.001
History of alcohol withdrawal	2.00	<0.001
History of drug abuse	1.70	<0.001
History of alcohol abuse	1.61	<0.001
Psychiatric history	1.31	<0.001
Traumatic brain injury	1.30	0.005
Male gender	1.12	0.02
Age	1.007	<0.001
Tobacco abuse	0.70	<0.001
Dementia	1.21	0.11

## Discussion

Our study on invasive medical devices, ubiquitous in health care, has yielded significant findings. Peripheral intravenous catheters, the most common among these devices, have a prevalence of up to 90% in inpatient care [4]. Other familiar devices include central venous catheters, urinary catheters, gastrostomy tubes, and nasogastric tubes. Our research highlights the importance of device-related complications as a patient safety issue, often used as indices of overall quality of care. While the discussion of adverse consequences of invasive devices commonly centers on infection, particularly central line-associated bloodstream infection (CLABSI) and catheter-associated urinary tract infection (CAUTI) [4], our study reveals that unplanned self-extraction of medical devices and their consequences may also cause harm.

For this study, we focused on devices other than endotracheal tubes. Endotracheal tube removal is specific to mechanical ventilation in that it is closely associated with sedation and weaning. These unique characteristics make it less valuable to compare directly to other devices. While self-extubation is the best-studied example of device self-extraction, different devices are also vulnerable [5]. In a study of potential consequences of excessive nursing workload, Carlesi et al. reported the self-removal of various devices at a rate of 5.5 per 1000 hospital days [6], similar to our results.

The unplanned self-removal of invasive devices can lead to serious patient harm. When a device is dislodged, its function is lost, often interrupting therapy. For instance, a nasoenteral tube used for postpyloric feeding of patients at high risk of aspiration may be technically challenging to place, leading to delayed feeding and potential malnutrition [7]. Moreover, replacing the device exposes the patient to procedural complications. Roubenoff and Ravich reported a series of four pneumothoraces due to misplacement of feeding tubes into the airway [8], underscoring the need for preventive measures to avoid such serious consequences.

Patients may suffer tissue trauma due to forcible removal of devices mechanically secured in place. Indwelling urinary catheters can cause bleeding and urethral damage if removed without deflating the balloon [9]. Removing a device without applying a proper seal may leave a breach in the skin or an internal structure, which may cause morbidity. Dreifuss and Silberzweig reported fatal exsanguination after an inadvertent central venous catheter removal [10]. Hasany et al. described a case of cerebral air embolism following self-extraction of a central line [11]. Premature removal of percutaneous endoscopic gastrostomy (PEG) tubes is a well-documented cause of peritonitis with the need for laparotomy [12].

Identifying high-risk patients allows us to direct attention and resources to prevent harm. Providers can respond with increased vigilance. Sitters have been used to detect and deter patient attempts to pull devices, although proof of efficacy is lacking [13]. Alarms have been attached to nasogastric tubes to alert providers to patient manipulation before the loss of the tube [14]. Physical and chemical restraints are often employed in the maintenance of indwelling devices, although, as with sitters, evidence of their efficacy is sparse. These measures remain common in clinical practice and are often viewed as undesirable but a practical necessity [15].

Devices may be mechanically secured. Dahlseng et al. reported significantly fewer dislodgement-related complications with PEG tubes following the implementation of a technique using T fasteners [16]. Nasogastric tubes can be bridled to make extraction more difficult. A single institution achieved an 80% reduction in unintentional nasointestinal tube loss following the implementation of a routine bridling protocol. Extrapolation of their findings predicted that nationwide implementation in Britain would result in 1,422 fewer cases of pneumonia or pneumothoraces and 768 fewer deaths [17].

Identification of risk factors permits attention to those that are modifiable. Our study reveals the central role of delirium in device self-removal. It was the most significant risk factor, with over three times the odds of device removal in delirious patients, suggesting that reducing delirium would also make devices more secure. Indwelling devices are risk factors for delirium [18,19]. Delirium, in turn, is a risk factor for unplanned device removal [3,20]. Restraints, both chemical and physical, may be used to protect the device, but restraints have adverse consequences and are themselves a risk factor for delirium [15].

Our study also showed that restraints and sitters are risk factors for SRMD. The direction of causality here is difficult to discern. It is unclear whether the restraints and sitters are applied due to agitation or are the cause of the agitation [21]. There may be a synergy between these factors, leading to a vicious circle in which measures to protect a device paradoxically make it more vulnerable by increasing delirium. Studies describing the management of ICU delirium show that it is very much a modifiable risk factor. Measures to reduce delirium, including restoration of a regular sleep/wake cycle, exposure to natural light via windows, access to hearing aids and glasses, and avoiding benzodiazepines, can all help protect devices [21].

Devices can be considered modifiable risk factors. A device not present cannot be inappropriately removed. Evidence shows that specific invasive devices are overused, and their removal may improve patient outcomes. In our study, nasogastric tubes represent half of all devices removed, which may be because nasogastric tubes are uncomfortable and challenging to conceal. Rao et al. showed that routine nasogastric decompression after elective colon surgery, while once the standard of care, showed no benefit in the studied population [22]. Public reporting and financial penalties have made device-related complications such as CAUTI and CLABSI expensive for the institutions responsible, which has led to campaigns to reduce their use, which can serve as a model for eliminating unnecessary devices. Rosenthal et al. showed that using indwelling urinary catheters could be reduced, lowering the CAUTI rate while avoiding the potential adverse effect of acute kidney injury [23]. Device use and the morbidity of unplanned removal may be reduced by carefully considering the indication for placement, removing devices as soon as possible, and not maintaining devices merely for convenience.

Our study showed that unplanned device removal was the strongest predictor of mortality of all binary risk factors examined. While it seems improbable that device self-removal is commonly the direct cause of mortality, it is more likely a marker of greater exposure to devices. Sicker patients need more tubes and catheters, which are vulnerable to self-extraction. However, our findings also point to a logical target for improvement. By reducing harm-related factors, we can significantly improve patient outcomes and the overall quality of care.

Limitations

These data come from an extensive public registry. While many comparisons will be statistically significant, deciding which are clinically meaningful requires careful judgement and expertise.

## Conclusions

Self-extraction of devices other than endotracheal tubes is a common occurrence in the ICU, affecting a broad spectrum of vulnerable patients. The risk is not limited to specific demographics or ICU subspecialties, with language barriers, tobacco use, and pain levels showing minimal correlation. The most critical risk factors are acute mental status changes rather than chronic conditions. Delirium emerges as a central concern. As a modifiable risk factor, it should be the primary focus of intervention. Recognizing and treating delirium is a crucial step that can help healthcare providers break the cycle of protecting devices with measures that may inadvertently increase delirium and, paradoxically, the risk of premature device removal. Device self-extraction is a significant risk factor for mortality. Even if unplanned extraction is not the primary cause of mortality, we can mitigate harmful associations with device self-extraction by proactively removing unnecessary devices.
